# Expression of E-cadherin and β-catenin in basaloid and conventional squamous cell carcinoma of the oral cavity: are potential prognostic markers?

**DOI:** 10.1186/1471-2407-14-395

**Published:** 2014-06-03

**Authors:** João Adolfo Costa Hanemann, Denise Tostes Oliveira, Suely Nonogaki, Inês Nobuko Nishimoto, Marina Lara de Carli, Gilles Landman, Luiz Paulo Kowalski

**Affiliations:** 1 Department of Clinic and Surgery, School of Dentistry, Alfenas Federal University, 700. CEP 37130-000 Alfenas, MG, Brazil; 2 Department of Stomatology-Area of Pathology, Bauru Dental School, São Paulo University, 17012-901 Bauru, São Paulo, Brazil; 3 Pathology Center, Adolfo Lutz Institute, 01246-000 São Paulo, Brazil; 4 Department of Head and Neck Surgery and Otorhinolaryngology, Cancer Hospital AC Camargo, 01509-010 São Paulo, Brazil; 5 Department of Pathology, Cancer Hospital AC Camargo, 01509-010 São Paulo, Brazil

**Keywords:** Carcinoma, Squamous Cell, Cadherins, Beta-Catenin, Prognosis, Immunohistochemistry

## Abstract

**Background:**

Basaloid squamous cell carcinoma presents with a preference for the head and neck region, and shows a distinct aggressive behavior, with frequent local recurrences, regional and distant metastasis. The alterations in the cadherin-catenin complex are fundamental requirements for the metastasis process, and this is the first study to evaluate the immunostaining of E-cadherin and β-catenin in oral basaloid squamous cell carcinoma.

**Methods:**

Seventeen cases of this tumor located exclusively in the mouth were compared to 26 cases of poorly differentiated squamous cell carcinoma and 28 cases of well to moderately differentiated squamous cell carcinoma matched by stage and tumor site. The immunostaining of E-cadherin and β-catenin were evaluated in the three groups and compared to their clinicopathological features and prognosis.

**Results:**

For groups poorly differentiated squamous cell carcinoma and basaloid squamous cell carcinoma, reduction or absence of E-cadherin staining was observed in more than 80.0% of carcinomas, and it was statistically significant compared to well to moderately differentiated squamous cell carcinoma (p = .019). A strong expression of β-catenin was observed in 26.9% and 20.8% of well to moderately differentiated squamous cell carcinoma and poorly differentiated squamous cell carcinoma, respectively, and in 41.2% of basaloid squamous cell carcinoma. The 5-year and 10-year overall and disease-free survival rates demonstrated no significant differences among all three groups.

**Conclusions:**

The clinical and biological behavior of three groups of the oral cavity tumors evaluated are similar. E-cadherin and β-catenin immunostaining showed no prognostic value for basaloid and conventional squamous cell carcinomas.

## Background

The basaloid squamous cell carcinoma (BSCC) is considered an aggressive variant from the squamous cell carcinoma, initially identified and meticulously described in head and neck area by Wain et al. [1]. This malignant neoplasm presents a predilection for the upper aerodigestive tract, although it can be present in other locations such as the lungs, esophagus, anal channel, and uterus [2,3]. In the mouth, these tumors have been identified in the floor of the mouth [4,5], tongue [5], buccal mucosa [6,7], gingiva [5,7], retromolar trigone [8], soft palate and oropharynx [2].

In head and neck area, BSCC is considered aggressive based mainly on clinical behavior, with frequent local recurrences, as well as regional and distant metastasis [9]. Some studies compared the clinical and/or biological evolution of this malignant neoplasm and of the conventional squamous cell carcinoma (SCC) [10,11]. However, the determination of the aggressive biological behavior of the SCC of head and neck has been based not only on the evaluation of the clinical aspects but mainly on the potential of cellular proliferation and tissue invasion, as well as on the loss of the expression of the molecules of adhesion of the epithelial neoplastic cells.

The alterations in the molecules of cellular adhesion in the SCC, mainly those associated with the cadherin-catenin complex [12-14] are fundamental requirements for the metastasis process, determining a more aggressive biological behavior and an unfavorable clinical evolution for these tumors [15]. The influence of the altered expression of the adhesion molecules in the clinical tumoral behavior of conventional SCCs with different degrees of cellular differentiation is an aspect frequently investigated [15-21]; however, to the best of our knowledge, the immunohistochemical expression of E-cadherin and β-catenin in oral BSCCs has not been reported. Thus, the purpose of this study was to assess the expression of E-cadherin and β-catenin in oral BSCCs, well to moderately differentiated SCCs (W/MSCC) and poorly differentiated SCCs (PDSCC), and compare the immunostaining of the two markers to their clinicopathological features and prognosis.

## Methods

### Patients

The oral BSCCs, W/MSCCs and PDSCCs used in this study are from the sample bank of the Pathology and Head and Neck Surgery and Otorhinolaryngology Departments of A. C. Camargo Cancer Hospital, diagnosed between 1970 and 2000. The 17 BSCCs and 26 PDSCCs have been previously analyzed by Sampaio-Goes et al. [22], and the 28 W/MSCCs were matched by clinical stage and tumor site with PDSCCs. All patients included in this retrospective study underwent surgical treatment of the primary oral carcinoma and postoperative radiotherapy and/or chemotherapy. The inclusion criteria were (1) diagnosis of SCC of the tongue, floor of the mouth, inferior gingiva, or retromolar area confirmed by biopsy; (2) patients not previously treated for any neoplasm; and (3) curative surgery as the first treatment. Exclusion criteria were (1) inoperable disease or unresectable tumors; (2) distant metastases at the time of admission; (3) presence of other primary tumors; (4) patients who refused surgical treatment.

The patients’ data were collected from the medical records and included sex, age, ethnic group, tobacco and/or alcohol consumption, tumor location and size, evidence of nodal metastases, T and N stage, treatment (surgery, postoperative radiotherapy, and/or adjuvant chemotherapy), and clinical follow-up (recurrence, occurrence of a second primary tumor, and death).

Cases were included in this study after three pathologists (JACH, DTO, GL) reevaluated the hematoxylin-eosin and periodic acid-Schiff (PAS) - stained sections from excised primary tumor specimens. Histopathological analysis was performed on the invasive tumor front in all cases. The tumor front was defined as the most advanced 3-6 tumor cell layers of a given tumor according to Piffko et al. [23]. Histopathological malignancy grading of the invasive tumor front was performed according to criteria described by Bryne et al. [24].

The study was approved by the Research Ethics Committee of A. C. Camargo Cancer Hospital (protocol #497/03). The authors read the Helsinki Declaration and followed the guidelines in this investigation.

### Immunohistochemistry

Formalin-fixed, paraffin-embedded 3-μm sections were cut from the oral BSCCs and SCCs for immunohistochemistry analysis with monoclonal anti-E-cadherin (36 clone, BD Transduction, ref C20820, Franklin Lakes, USA) and anti-β-catenin (14 clone, BD Transduction, ref C19220, Franklin Lakes, USA). Immunohistochemical staining was performed using a standard streptavidin-biotin-peroxidase complex method (StreptABComplex/HRP, Duet Mouse/Rabbit, Dako ref K0492, Glostrup, Denmark). After antigen retrieval with 10 m*M* citrate buffer, pH 6.0, in a pressure cooker, endogenous peroxidase activity was blocked by incubation in 3% H_2_O_2_ for 20 minutes. The sections were incubated overnight at 4°C with the following primary antibodies and dilutions: E-cadherin (1:700) and β-catenin (1:1000) in bovine serum albumin (BSA) solution to block a nonspecific reaction. The antigen - antibody reaction was visualized using 3’3 diaminobezidine tetrahydrochloride 60 mg% (DAB/SIGMA, ref D-5637, St. Louis, MO). Sections were counterstained with Harris hematoxylin before being dehydrated and cover slipped. Dermatofibroma and a fragment of intestine served as positive control for E-cadherin and β-catenin, respectively. For a negative control, the primary antibody was omitted during the immunohistochemical staining.

### Immunostaining quantification

For each case, 10 non-overlapping fields (107.040.116 μm^2^) at 400X magnification of invasive tumor front were digitally captured with a Samsung camera attached to a light microscope (Axioskop2 plus, Zeiss, Jena, Germany) and recorded by *Image Lab* computer program system. The images in each slide of E-cadherin and β-catenin antibodies were assessed by semiquantitative score method as previously instituted in oral BSCC and SCC by Sampaio-Goes et al. [22], based on the sum of the proportion of immunopositive tumor cells, the intensity of the expression of markers used and cellular location (membranous or cytoplasmic and nuclear) of immunostaining.

The rate of positive tumor cells was divided into three groups:

• **0** = ≤ 10% of positive tumor cells;

• **1** = 11 a 50% of positive tumor cells;

• **2** = >50% of positive tumor cells.

The intensity of immunostaining was evaluated as follows:

• **0** = without immunostaining;

• **1** = weak immunostaining;

• **2** = strong immunostaining.

The cellular location of immunostaining was divided into three groups:

• **0** = without immunostaining;

• **1** = cytoplasmic and nuclear immunostaining;

• **2** = membranous immunostaining.

The sum of the scores, based on the proportion, the intensity and cellular location of E-cadherin and β-catenin was classified as:

• **0**–**4** = Score 1 = absent or weak immunostaining;

• **5**–**6** = Score 2 = strong immunostaining.

Immunostaining results were evaluated by three investigators (JACH, GL, DTO) without prior knowledge of the tumor's histopathologic features and the patient's clinical status. The cases of incongruence were reviewed for a final consensus.

### Statistical analysis

First, to verify the differences between the mean ages, the Student’s *t*-test was used and, subsequently, the age variable was categorized. The Fisher exact test or chi-square test was used to assess the association between demographic, clinical and microscopic variables, with a significance level of 5%.

Survival rates (overall and disease-free survival), assessed at 5 and 10 years, were calculated by the Kaplan - Meier method. Comparisons among survival curves were verified by the log-rank test with a significance level of 5%.

## Results

The clinical and demographic parameters of patients with oral BSCC and conventional oral PDSCC or W/MSCC were similar, as summarized in Table 1.

**Table 1:** Distribution of the clinical features of patients with oral BSCC, W/MSCC and PDSCC

**Clinical features**	**No. of cases (%)**						
	**BSCC**		**PDSCC**		**W/MSCC**		***p*** **value** *
Sex							
Male	15	88.2	25	96.1	26	92.9	NA
Female	2	11.8	1	3.9	2	7.1	
Ethnic group							
White	15	88.2	22	84.6	24	85.7	NA
Non-White	2	11.8	4	15.4	4	14.3	
Age, y							
≤ 59	7	41.2	15	57.7	16	57.1	.504
> 59	10	58.8	11	42.3	12	42.9	
Tobacco^#^							
Yes	12	85.7	22	91.7	22	88.0	NA
No	2	14.3	2	8.3	3	12.0	
Alcohol^#^							
Yes	10	76.9	21	87.5	22	88.0	NA
No	3	23.1	3	12.5	3	12.0	
Clinical T classification							
T1 - 2	5	29.4	6	23.1	9	32.1	.754
T3 - 4	12	70.6	20	76.9	19	67.9	
Clinical N classification							
N0	5	29.4	8	30.8	9	32.1	.981
N1 - 2 - 3	12	70.6	18	69.2	19	67.9	
Tumor site							
Tongue	3	17.7	5	19.3	7	25.0	NA
Floor of mouth	10	58.8	9	34.6	8	28.6	
Retromolar/gingiva	4	23.5	12	46.1	13	46.4	
Neck dissection							
Without	0	0.0	0	0.0	1	3.6	NA
Ipsilateral	9	53.0	19	73.0	22	78.6	
Bilateral	8	47.0	7	27.0	5	17.8	
Radiotherapy							
Yes	15	88.2	22	84.6	18	64.3	NA
No	2	11.8	4	15.4	10	35.7	
Chemotherapy							
Yes	3	17.7	7	26.9	5	17.9	NA
No	14	82.3	19	73.1	23	82.1	
Total	17	100	26	100	28	100	

Abbreviations: *BSCC* basaloid squamous cell carcinoma, *PDSCC* poorly differentiated squamous cell carcinoma, *W/MSCC* well to moderately differentiated squamous cell carcinoma, *NA*not applicable.

*Analysis by chi-square test at 5% significance level.

^#^Excluding patients with lost records.

**Figure 1:**
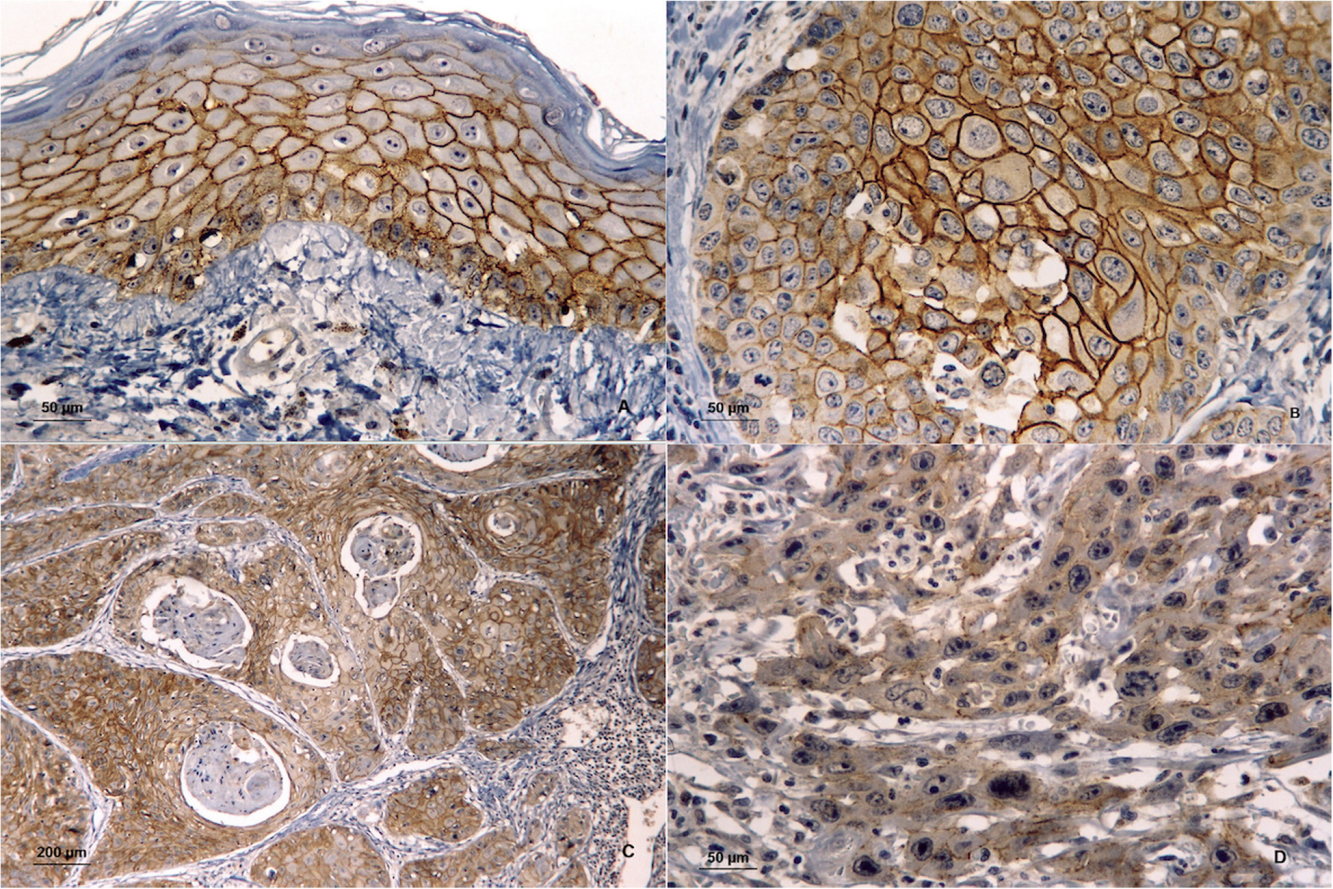
**Immunostaining for E-cadherin. A)** Immunostaining for E-cadherin in normal oral mucosa. **B)** Strong membranous immunostaining in the oral basaloid squamous cell carcinoma (BSCC). **C)** Most neoplastic cells of well to moderately differentiated squamous cell carcinoma (W/MSCC) showed strong membranous immunopositivity. **D)** Poorly differentiated squamous cell carcinoma (PDSCC) showed weak membranous immunostaining. (A, B, C and D, immunohistochemistry E-cadherin; original magnifications, A, B and D, ×400; C, ×100).

Cytoplasmic and membranous E-cadherin expression was detected in the oral mucosa with a normal pattern and in the tumor cells. A strong predominately membranous expression of E-cadherin antibody was observed in more than 40% of W/MSCCs. In the PDSCC and BSCC groups, 19% and 12% of the specimens, respectively, showed a strong cytoplasmic immunostaining for E-cadherin (Table 2). Furthermore, in the BSCCs, the membranous expression of E-cadherin was observed in only a few rare tumors (Figure 1).

**Table 2:** Immunohistochemical analysis of patients with oral BSCC, W/MSCC and PDSCC

**Antibodies**	**No. of cases (%)**						
	**BSCC**		**PDSCC**		**W/MSCC**		***p*** **value** *
E-cadherin							
Weak immunostaining	15	88.2	21	80.8	15	53.6	.019
Strong immunostaining	2	11.8	5	19.2	13	46.4	
β-catenin							
Weak immunostaining	10	58.8	20	76.9	20	71.4	.355
Strong immunostaining	7	41.2	6	23.1	8	28.6	
Total	17	100	26	100	28	100	

Abbreviations: *BSCC* basaloid squamous cell carcinoma, *PDSCC* poorly differentiated squamous cell carcinoma, *W/MSCC* well to moderately differentiated squamous cell carcinoma.

*Analysis by chi-square test at 5% significance level.

**Figure 2:**
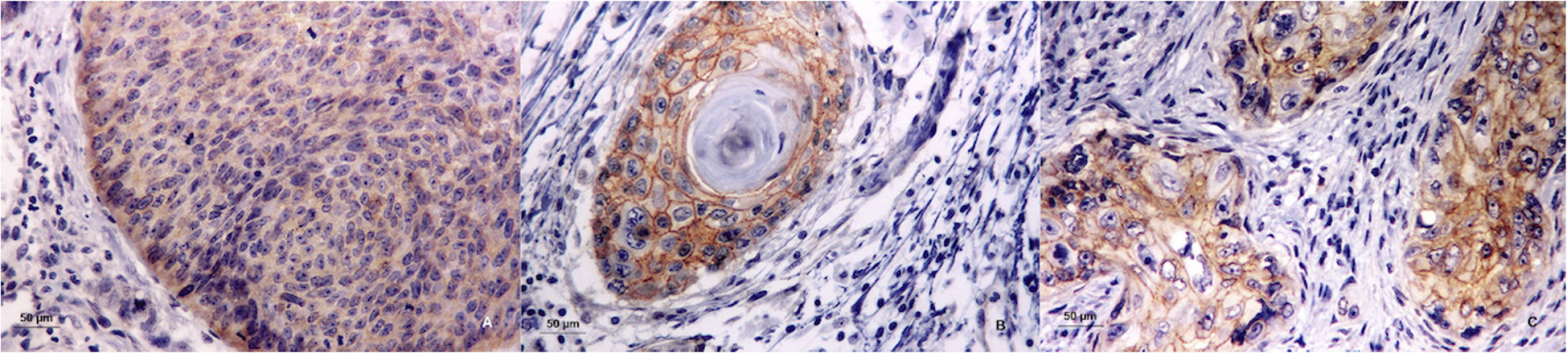
**Immunostaining for β-catenin. A)** Immunostaining for β-catenin in oral basaloid squamous cell carcinoma (BSCC) showed weak cytoplasmic and nuclear immunopositivity. **B)** Well to moderately differentiated squamous cell carcinoma (W/MSCC) showed strong membranous, cytoplasmic and nuclear immunopositivity. **C)** Poorly differentiated squamous cell carcinoma (PDSCC) showed weak membranous, cytoplasmic and nuclear immunostaining. (A-C, immunohistochemistry β-catenin; original magnifications, A-C, ×400).

In addition to E-cadherin, the expression of β-catenin showed nuclear, cytoplasmic and membranous immunostaining in tumor cells. Weak immunostaining for β-catenin was detected in over 70% of both groups of SCCs (PDSCC and W/MSCC) and in 59% of BSCCs. Strong immunostaining for β-catenin antibody was observed in only 28% and 23% of W/MSCC and PDSCC groups, respectively (Table 2). In BSCCs, membranous expression of β-catenin, although irregular, was detected along with cytoplasmic and nuclear expression in 41% of the specimens (Figure 2).

**Figure 3:**
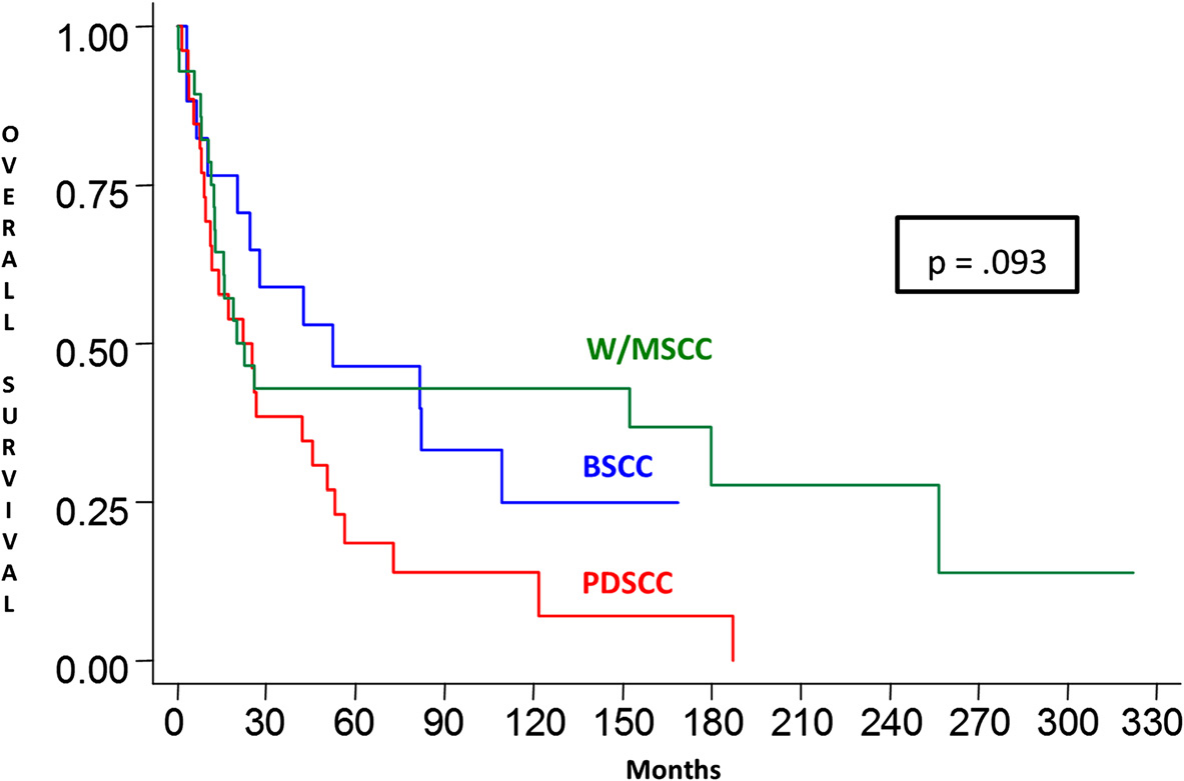
**Overall survival.** Kaplan-Meier overall survival curves showing no statistically significant differences between overall survival of patients with W/MSCCs, BSCCs and PDSCCs. W/MSCC, well to moderately differentiated squamous cell carcinoma; BSCC, basaloid squamous cell carcinoma; PDSCC, poorly differentiated squamous cell carcinoma.

**Figure 4:**
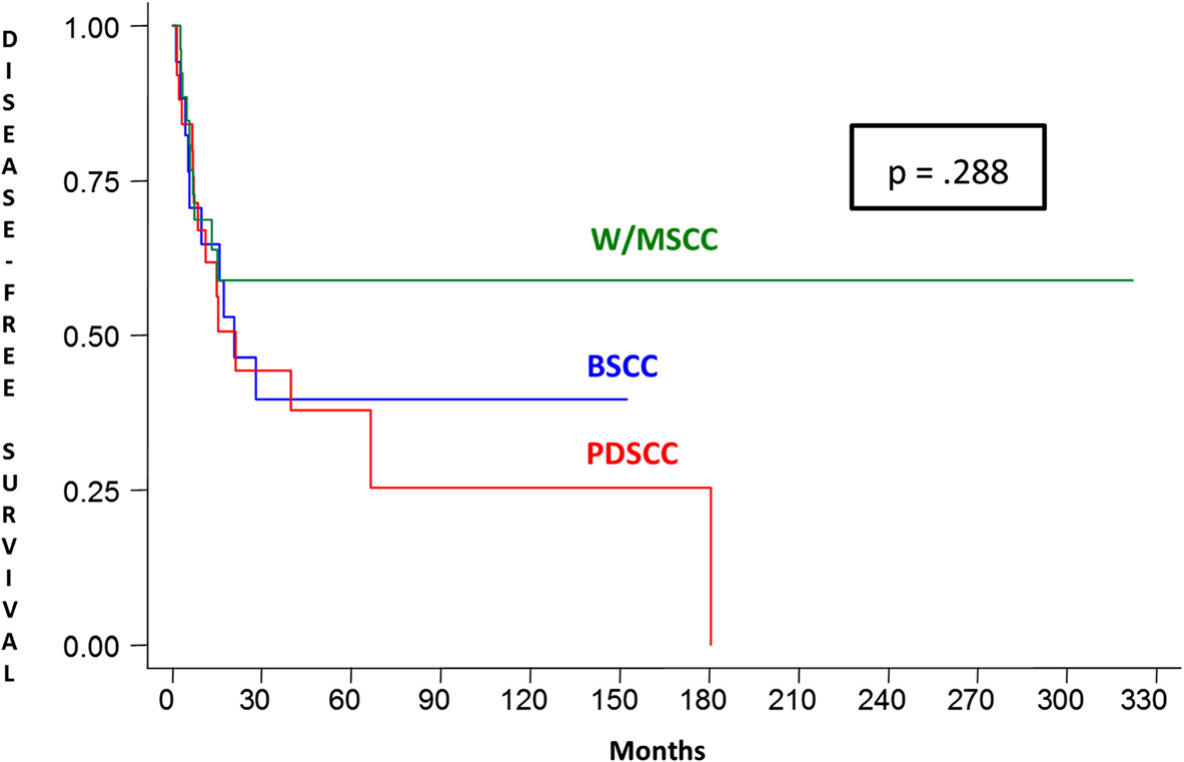
**Disease-free survival.** Kaplan-Meier disease-free survival curves showing no statistically significant differences between disease-free survival of patients with W/MSCCs, BSCCs and PDSCCs. W/MSCC, well to moderately differentiated squamous cell carcinoma; BSCC, basaloid squamous cell carcinoma; PDSCC, poorly differentiated squamous cell carcinoma.

A statistically significant difference in the expression of E-cadherin in tumor cells was observed in the W/MSCC group compared with BSCC and PDSCC groups (p = .019). However, no statistically significant difference was found between the three different groups of carcinomas studied with regard to epithelial expression of β-catenin (Table 2).In addition to clinically detected positive lymph nodes, no statistically significant correlation between microscopically confirmed positive lymph nodes (pN) and E-cadherin and β-catenin immunostaining was established in the three SCC groups studied. However, patients with positive lymph nodes at diagnosis had a twofold increase in the relative risk of death (p = .025). The comparison between the curves of overall survival and disease-free survival showed no statistically significant differences between BSCC, PDSCC and W/MSCC groups (Figures 3 and 4). Also there was no difference between the overall and disease-free survival rates, regarding immunostaining for E-cadherin and β-catenin, for all the patients.

## Discussion

BSCC has been considered an aggressive variant of SCC at different anatomic sites [7]; however, a comparative study [25] between these two neoplasias in the oral cavity have included samples with a small number of patients, which precludes reliable comparisons of the clinical and biological behavior between oral BSCC and SCC. The comparative analysis of the BSCCs, PDSCCs and W/MSCCs revealed demographic and clinical results similar to those described in the literature [26-28]; these tumors occurred mainly in male patients with a mean age of 58 years, being consumers of tobacco and alcohol.

Regarding the clinical behavior, we found that the highest rate of local recurrence occurred in the PDSCC group (38.5%) compared to BSCC and W/MSCC groups. The rate of local recurrence for the BSCC was 17.7%, which is similar to rates found by other authors [2,29]. A higher frequency of regional recurrence and distant metastasis was detected in the BSCC group than the PDSCC group, in agreement with Fritsch et al. [30], suggesting a more aggressive clinical profile for BSCC. The lungs were the most commonly affected site by both metastatic neoplasms. No patient of the W/MSCC group developed distant metastases, and the regional recurrence rate of this group was similar to that observed for the BSCC.

Besides the clinical and morphological characteristics of malignancy, the expression of cell adhesion molecules is fundamental to the design of the biological behavior of tumors. The cell adhesion molecules E-cadherin and β-catenin are responsible for the maintenance of intercellular unions associated with the process of tumor invasion and metastatic spread in SCCs. The reduction or loss of expression of the cadherin-catenin complex via mutations or loss of heterozygosity occurs frequently during carcinogenesis, affecting its tumor suppressor activity [12-14,31]. As the BSCC is considered a variant from the SCC [7], the role of E-cadherin and β-catenin in the pathogenesis of BSCC may be similar to SCC. Overall, the immunostaining of cadherins and catenins has been more clearly verified in well-differentiated SCCs, which maintain their cell adhesiveness and are less invasive compared with PDSCCs with an infiltrative invasion pattern and little or no cell cohesion [15,20]. E-cadherin showed weaker immunostaining for BSCC than W/MSCC, but there is no difference between the immunostaining for BSCC and PDSCC, suggesting that the BSCC may present the similar biological behaviour to PDSCC.

E-cadherin immunostaining compared to clinical features, such as TNM stage, tumor location, local and regional recurrence and distant metastasis, showed no statistically significant difference in tumor groups analyzed. However some authors [20] have associated loss or reduced expression of E-cadherin in SCCs with a higher frequency of regional metastases. Regarding β-catenin, loss or reduced expression was detected numerically in more than 70% of SCCs and in 59% of BSCCs; however, no statistically significant difference was detected when comparing the expression of this cell adhesion molecule between the three tumor groups analyzed. These results agree with those of Lopez-Gonzalez et al. [18] who also found a greater reduction in immunostaining for β-catenin in PDSCCs compared to W/MSCCs of the larynx.

Catenins play a critical role in controlling cell adhesion mediated by E-cadherin; this indicates that immunoreactivity does not always imply a normally functioning cadherin-catenin complex [14]. Therefore, the influence of these molecules in the process of invasion and metastasis should be analyzed together [15-17,19,21], as in this study.

Patients with BSCC, PDSCC and W/MSCC of the oral cavity in the present study showed, at diagnosis, an advanced clinical stage associated with a high frequency of regional lymph node involvement. Study variable N consisted of an independent factor indicative of unfavorable prognosis of the three tumor groups studied. Patients with positive lymph nodes at diagnosis had a twofold increase in the relative risk of death (p = .025), regardless of age, gender, tumor and immunoreactivity for E-cadherin and β-catenin. Despite the BSCC presented higher rates of distant metastases, curves of overall survival and disease-free survival showed no statistically significant differences between BSCC, PDSCC and W/MSCC groups, similar to Fritsch et al. [30], therefore oral BSCC demonstrated a comparable prognosis to conventional SCC.

## Conclusion

The results of this study do not verify that oral BSCCs have a more aggressive clinical and biological behavior than oral SCCs. E-cadherin and β-catenin immunostaining showed no prognostic value for oral BSCCs, PDSCCs and W/MSCCs matched by location and clinical stage.

## Competing interests

The authors declare that they have no competing interests.

## Authors’ contributions

JACH reevaluated all the cases that were included in the study and he was responsible for the acquisition and interpretation of data. DTO provided substantial contributions to the conception and design of the study. SN provided substantial contributions to the immunohistochemical staining and quantification. INN was responsible for the analysis and interpretation of data for the study. MLC was involved in drafting the manuscript and revised it critically for important intellectual content. GL was responsible for the diagnosis of all the cases and reassessed the cases to include them in the study. LPK performed the treatment of all the cases that were included in this study. All authors read and approved the final manuscript.

## Pre-publication history

The pre-publication history for this paper can be accessed here:

http://www.biomedcentral.com/1471-2407/14/395/prepub
